# Handbook Integrated Care: Amelung, V.E., Stein, V., Goodwin, N., Balicer, R., Nolte, E., Suter, E. (Eds.), Publisher: Sprinter International Publishing. DOI: 10.1007/978-3-319-56103-5

**DOI:** 10.5334/ijic.4672

**Published:** 2019-02-26

**Authors:** Mudathira Kadu

**Affiliations:** 1 University of Toronto, Institute of Health Policy, Management and Evaluation, CA

**Keywords:** integrated care, health care, social care, person-centered care, population-based approaches

Edited by Volker Amelung, authors Viktoria Stein, Nicholas Goodwin, Ran Balicer, Ellen Nolte and Esther Suter set out to bring together for the first time in one book, the evidence for key concepts, management elements and tools that have been implemented internationally to enhance integrated care. It specifically offers the reader fundamental building blocks of integrated care, illustrating a multitude of best-practice examples from around the world. The book offers a substantive knowledge translation tool, bringing together the growing evidence on essential approaches that have been shown to facilitate successful implementation of integrated care. Boasting 37 chapters and written by over 30 well-renowned experts in the field of integrated care, the book chapters range from the rationale behind integrated care, effective practices in the management of integrated care, evaluation and health service research, tailoring interventions, to various client groups and case studies of integrated care. Each chapter introduces the reader to the key concept, methodologically reviewing the literature and identifying gaps in evidence. This is further bolstered by supportive illustrations, case studies and summary boxes; offering the reader helpful visual learning and practical examples while reading each chapter.

To enable the reader to understand the fundamentals of integrated care, the book is divided into six key sections, with six chapters each. The preface provides a guiding framework for key topics covered in the book, walking readers through various dimensions of integrated care. The book sections are organized systematically to build upon each other. The first section of the Handbook provides a conceptual rationale for why integrated care is important. Careful effort is made to outline the varying definitions and characteristics of integrated care across health systems. The authors note that because integrated care has come to mean different things to different people, this lack of unifying concept has often challenged the development of commonly understood strategies to support its implementation. The reader leaves the first section of the book with a better understanding of the distinct dimensions of what integrated care looks like in practice and a clearer knowledge of the evidence behind integrated care.

The second and third sections of the book shed light into the different management aspects of integrated care that vary significantly across systems. The chapters methodologically outline the fundamental and vital elements of management in integrated care. Some of the different scopes of management discussed include: governance and accountability, financing and reimbursement, culture and vales, health service planning, leadership in integrated care, integrated care and the labour force and successful change management practices. Illustrations are provided to showcase how vital management practices interplay. The authors highlight this with an example of network-based integrated care models, which tend to be quite adaptive to healthcare context with hierarchal governance.

There is an increasing interest in evaluating various models of integrated care to determine whether they address patients’ needs and preferences. As such, the fourth section describes the fundamentals of evaluating complex interventions including economic evaluation, as they apply to integrated care. The authors seek to address the knowledge deficit of how the evaluation of integrated care can be used to answer questions such as *what works in integrated care, for whom and in what care context?* The evaluation of integrated care can be a challenging task that requires rigorous methods and scientific approaches. Effort is taken in the Handbook to build a case for the importance of evaluation in evidence-based decision making and in quality improvement. The authors describe the unique obstacles to performing evaluations of integrated care interventions, discuss methods and techniques that can be used to address them, and set the basis to develop a research agenda for health economics in integrated care.

The potential for integrated care to be adapted for select patient groups is analyzed in the fifth section of the Handbook, which assists in elevating the practical applicability of the book. The section showcases design and implementation concepts for fields such as geriatrics, pediatrics, mental health, end-of-life and care for rare diseases. In line with the goals of the Handbook, these illustrations strike a balance between the theoretical discourse, conceptual design and practical examples. While integrated care as a concept is applicable to any system, the model, tools and instruments must be contextualized; as different system environments require different approaches for implementation.

Perhaps the book’s most exciting chapters are in the sixth and final section, where the authors demonstrate examples from eight distinct health systems. These include: Canada, Germany, Scotland, the United States of America, Switzerland, The Netherlands, New Zealand and Israel. The goal of the final chapters is not to reinvent the wheel with integrated care, but rather to learn from the experience of other countries. The book concludes with the recognition that successful integrated care programs are often a mosaic of ideas and concepts from a variety of settings and disciplines, woven together in a way that is most adaptable to the context in which it is being implemented.

Integrated care in the last few years has evolved into a dominant topic in healthcare management and health system design. A wide range of approaches and models have been developed and implemented, as shown by the plethora, and sometimes fragmented literature on this topic. The Handbook provides an excellent starting point for those exploring various dimensions of integrated care. A potential limitation of this book is that despite the vast international case studies examined, the reader might notice a dearth of information on the implementation of integrated care in low to middle income countries. More specifically, the evaluation of policy changes and strategies to implement integrated care in low income countries. For example, examining how integrated care can be used to address issues such as infectious diseases, would have expanded the focus of the book. Ultimately, in a field where the information is often dispersed, many readers might view the book as an ‘integrated care bible’, finding helpful the wealth of information the Handbook has to offer. The target audience of this book can include healthcare decision-makers seeking policy inspirations from other health system contexts, and clinicians and health practitioners hoping to learn from best-practice examples. Others who may find this Handbook helpful are health service leaders and researchers interested in understanding the evidence for integrated care programs; particularly what works for whom, how and under which enabling conditions.

